# Blood myxovirus resistance protein‐1 measurement in the diagnostic work‐up of suspected COVID‐19 infection in the emergency department

**DOI:** 10.1002/iid3.609

**Published:** 2022-03-29

**Authors:** Kirby Tong‐Minh, Samantha van Hooijdonk, Marjan A. Versnel, Cornelia G. van Helden‐Meeuwsen, Petrus Martin van Hagen, Eric C. M. van Gorp, Henrik Endeman, Yuri van der Does, Virgil A. S. H. Dalm, Willem A Dik

**Affiliations:** ^1^ Department of Emergency Medicine Erasmus University Medical Center Rotterdam The Netherlands; ^2^ Department of Immunology Erasmus University Medical Center Rotterdam The Netherlands; ^3^ Department of Internal Medicine Section of Allergy & Clinical Immunology, Erasmus University Medical Center Rotterdam The Netherlands; ^4^ Department of Viroscience Erasmus University Medical Center Rotterdam The Netherlands; ^5^ Department of Intensive Care Erasmus University Medical Center Rotterdam The Netherlands; ^6^ Laboratory Medical Immunology, Department of Immunology Erasmus University Medical Center Rotterdam The Netherlands

**Keywords:** biomarker, COVID‐19, emergency department, MxA

## Abstract

**Introduction:**

Myxovirus resistance protein 1 (MxA) is a biomarker that is elevated in patients with viral infections. The goal of this study was to evaluate the diagnostic value of MxA in diagnosing COVID‐19 infections in the emergency department (ED) patients.

**Methods:**

This was a single‐center prospective observational cohort study including patients with a suspected COVID‐19 infection. The primary outcome of this study was a confirmed COVID‐19 infection by RT‐PCR test. MxA was assessed using an enzyme immunoassay on whole blood and receiver operating chart and area under the curve (AUC) analysis was conducted. Sensitivity, specificity, negative predictive value, and positive predictive value of MxA on diagnosing COVID‐19 at the optimal cut‐off of MxA was determined.

**Results:**

In 2021, 100 patients were included. Of these patients, 77 patients had COVID‐19 infection and 23 were non‐COVID‐19. Median MxA level was significantly higher (*p* < .001) in COVID‐19 patients compared to non‐COVID‐19 patients, respectively 1933 and 0.1 ng/ml. The AUC of MxA on a confirmed COVID‐19 infection was 0.941 (95% CI: 0.867–1.000). The optimal cut‐off point of MxA was 252 ng/ml. At this cut‐off point, the sensitivity of MxA on a confirmed COVID‐19 infection was 94% (95% CI: 85%–98%) and the specificity was 91% (95% CI: 72%–99%).

**Conclusion:**

MxA accurately distinguishes COVID‐19 infections from bacterial infections and noninfectious diagnoses in the ED in patients with a suspected COVID‐19 infection. If the results can be validated, MxA could improve the diagnostic workup and patient flow in the ED.

## Graphical abstract

Elevated myxovirus resistance protein‐1 (MxA) levels accurately distinguish COVID‐19 infections from bacterial infections and noninfectious diagnoses in patients with suspected COVID‐19 infection in the emergency department. MxA measurements may be of added value in the diagnostic workup and patient flow in the emergency department.

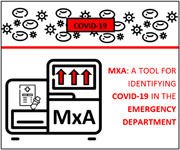

## 1 INTRODUCTION

Coronavirus disease (COVID‐19) caused by the novel Coronavirus (SARS‐CoV‐2) causes a high burden on hospital capacities worldwide, especially at the emergency department (ED).^1^ Patients with a suspected COVID‐19 infection are initially examined and evaluated at the ED. Diagnosing COVID‐19 at an early stage in the ED is important because patients with a confirmed COVID‐19 infection need to be isolated and may require treatment with immunomodulatory medication.^2^ When a COVID‐19 infection is ruled out, patients may require antibiotic treatment. However, rapid identification of COVID‐19 infections in the ED is challenging. The gold standard for diagnosing COVID‐19 is reverse transcriptase‐polymerase chain reaction (RT‐PCR), which may take up to 24 h before the result is available. Rapid antigen tests are available, but have a lower sensitivity in detecting SARS‐CoV‐2 than RT‐PCR and are not widely used in ED.^3^ Vital signs and routine laboratory tests, such as white blood cell count (WBC) and C‐reactive protein (CRP), are often insufficient for diagnosing COVID‐19 in ED.^4^ Different biomarkers have been investigated as a predictor of COVID‐19 disease, including procalcitonin and interleukin‐6. These biomarkers are accurate predictors of disease severity in COVID‐19, but have only limited added value in the diagnosis of COVID‐19 in the ED.^5^, ^6^ Myxovirus resistance protein 1 (MxA) is a key protein in the interferon (IFN) type‐1‐regulated antiviral response.^7^ MxA measurements in full blood were shown to distinguish viral infections from other types of infections in ED.^8^ Therefore, it is likely that MxA will also be elevated in COVID‐19 infections. MxA may represent a valuable biomarker in the diagnostic workup in the ED, especially when results are generated more rapidly than the currently used RT‐PCR.

The goal of this study is to evaluate the diagnostic value of whole blood MxA in identifying COVID‐19 infections in ED patients.

## 2 METHODS

This study is part of the PIAC‐19 study, a single‐center prospective observational cohort study. The study was conducted at the Laboratory Medical Immunology and ED of Erasmus University Medical Center, an academic hospital with annually 40.000 ED visits. The study was approved by the local institutional review board and registered under number: NL73846.078.20.

Inclusion criteria were: referral because of clinical suspicion of COVID‐19 infection, age ≥18 years, and written informed consent. Exclusion criteria were: insufficient knowledge of the Dutch language, non‐COVID‐19‐related incapacitated subjects, or absence of informed consent.

Suspected COVID‐19 infection was defined as patients presented at the ED with any of the following symptoms: dry cough, fever, headache, diarrhea, dyspnea, rhinitis, or lack of taste or scent.

The sample size consisted of a convenience sample. Due to the availability of the research personnel, screening for eligibility and enrollment of patients took place from Monday to Friday during working hours. Any patient visiting the ED was screened for eligibility during this period.

### 2.1 Data collection

Patient data including demographics, comorbidities, duration of symptoms, vital signs, and laboratory tests were collected during the ED visit. The use of immunosuppressive medication, defined as the use of systemic corticosteroids >7.5 mg prednisone equivalent per day, use of disease‐modifying antirheumatic drugs (DMARDs), biologicals, or anti‐rejection medication after organ transplantation, was recorded. Standard laboratory testing during the ED visit included white blood cell count, CRP, procalcitonin, lactate, ferritin, and lactate dehydrogenase (LDH). Patients were followed up for 30 days after hospital discharge.

After obtaining informed consent, extra blood was drawn in a sodium heparin tube. MxA enzyme immunoassay was conducted as previously described.^9^ Briefly, 25 μl of the heparinized blood was lysed 1:20 in hypotonic buffer containing 1.5% bovine serum albumin (BSA), 1% ascorbic acid, 0.5% NaHCO3%, and 0.05% NaN3. Diluted specimens were frozen and stored at −80°C until assayed. MxA was measured in batch. Fifty microliters of thawed lysed whole blood samples and biotinylated detector‐monoclonal antibody (MAb) were added in duplicate to MAb‐coated microtitre strips and subsequently incubated for 2 h at room temperature under constant shaking. Streptavidin–peroxidase and tetramethylbenzidine peroxidase substrate solution were used for detection. Absorbance was measured at 450 nm and MxA levels were quantified from a standard curve. The lower limit of detection for MxA was 10 μg/L. MxA levels were not available to the treating physician.

### 2.2 Primary outcome

The primary outcome of this study was a confirmed COVID‐19 infection by RT‐PCR test. An RT‐PCR test was part of the standard clinical workup at the ED and ordered by the treating physician. Patients were classified as COVID‐19 patients when the RT‐PCR test was positive and non‐COVID‐19 when the RT‐PCR test was negative.

### 2.3 Statistical analysis

Normally distributed variables were reported as mean with standard deviation (SD), non‐normally distributed variables as median with interquartile range (IQR). Cases with missing data that were not used in the primary or secondary analyses were deleted

Differences in dichotomous variables between the confirmed COVID‐19 and non‐COVID‐19 patients were analyzed with a *χ* ^2^ test. Differences in continuous variables were analyzed using an independent sample *t* test for normally distributed data and a Mann–Whitney *U* test for nonnormally distributed data.

For the primary outcome, we calculated a receiver operating chart (ROC) and calculated the area under the curve (AUC) using MxA as continuous variable. With the AUC, we calculated the optimal cut‐off point of MxA using the Youden's Index.^10^ Following, we used this cut‐off point to calculate the sensitivity and specificity, negative predictive value, and positive predictive value of MxA on diagnosing COVID‐19.

For the secondary analysis, we used the cut‐off point of 20 and 423 ng/ml, as previously described in literature, and calculated the sensitivity and specificity of MxA on diagnosing COVID‐19.^11^, ^12^ Furthermore, we tested the difference in MxA level between patients using immunosuppressive medication and with an immunodeficiency.

Statistical analyses were performed using “R” version 4.1.0.

## 3 RESULTS

**Figure 1:**
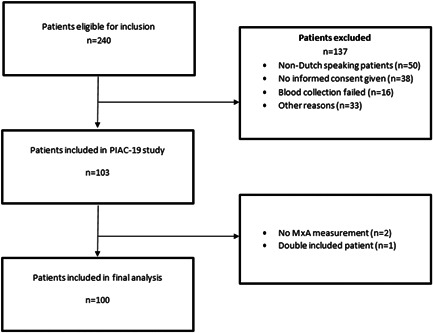
Flowchart of included patients. MxA, myxovirus resistance protein‐1

Between March and June 2021, a total of 240 patients were eligible for inclusion, of which 100 patients were included in this study (Figure 1). Of these patients, 77 (77%) patients had a COVID‐19 infection and 23 (23%) were non‐COVID‐19. Of the 23 non‐COVID‐19 patients, 11 (11%) had a bacterial infection and 12 (12%) had a noninfectious alternative diagnosis. There were missing data in PCT (16%), ferritin (15%), and LDH (3%). The cases with these missing data were deleted in the analyses of the baseline characteristics.

Baseline characteristics are shown in Table 1. There was no significant difference between the COVID‐19 patients and non‐COVID‐19 patients in demographic data and comorbidities. COVID‐19 patients had a lower heart rate than non‐COVID‐19 patients (*p* = .021). Other vital parameters were not significantly different between groups. In laboratory parameters, ferritin (*p* = .001), LDH (*p* < .001), and WBC (*p* < .045) were higher in COVID‐19 patients. COVID‐19 patients were admitted to the general ward more often (*p* = .001) and required ICU admission more often (*p* < .001). The mortality rate in COVID‐19 patients was higher than in non‐COVID‐19 patients (*p* < .001).

**Table 1:** Baseline characteristics

Patient characteristics		All patients	COVID‐19	Non‐COVID‐19	*p*
		*n* = 100	*n* = 77	*n* = 23	
Demographic data					
Sex: male	*n* (%)	62 (62)	49 (63.6)	13 (56.5)	0.71
Age	Median (IQR)	59 (24)	62 (27)	58 (23)	0.614
Comorbidity: pulmonary disease	*n* (%)	27 (27)	20 (26)	7 (30.4)	0.877
Comorbidity: cardiovascular disease	*n* (%)	45 (45)	34 (44.2)	11 (47.8)	0.943
Comorbidity: diabetes mellitus	*n* (%)	18 (18)	14 (18.2)	4 (17.4)	1
Comorbidity: malignancy	*n* (%)	21 (21)	13 (16.9)	8 (34.8)	0.113
Comorbidity: renal disease	*n* (%)	15 (15)	11 (14.3)	4 (17.4)	0.974
Comorbidity: auto‐immune diseases	*n* (%)	15 (15)	11 (14.3)	4 (17.4)	0.974
Comorbidity: immunodeficiency	*n* (%)	11 (11)	7 (9.1)	4 (17.4)	0.461
Comorbidity: central nervous system diseases	*n* (%)	11 (11)	8 (10.4)	3 (13)	1
Immunosuppressive drug use	*n* (%)	46 (46)	36 (46.8)	10 (43.5)	0.782
Vital parameters					
Heartrate (/min)	mean (SD)	86 (16)	84 (14)	94 (16)	0.021
Respiratory rate (/min)	median (IQR)	18 (3)	18 (4)	16 (8)	0.422
Oxygen saturation (%)	median (IQR)	96 (3)	96 (3)	96 (1)	0.849
Diastolic blood pressure (mmHg)	mean (SD)	78 (11)	78 (11)	76 (13)	0.51
Systolic blood pressure (mmHg)	mean (SD)	132 (17)	132 (17)	133 (19)	0.877
Temperature (celsius)	mean (SD)	37.6 (1)	37.6 (1.1)	37.6 (1)	0.492
Laboratory testing					
Procalcitonin (ng/ml)	median (IQR)	0.11 (0.19)	0.11 (0.16)	0.14 (0.19)	0.831
CRP (mg/L)	median (IQR)	63 (98)	63 (94)	46 (135)	0.632
Leucocyte count	median (IQR)	6.4 (4.3)	5.9 (3.7)	7.6 (5.6)	0.045
Ferritine (µg/ml)	median (IQR)	677 (855)	798 (973)	310 (603)	0.001
LDH (U/L)	median (IQR)	316 (139)	338 (129)	243 (86)	<0.001
Lactate (mmol/L)	median (IQR)	1.3 (0.8)	1.3 (0.8)	1.2 (1.3)	0.764
MxA (ng/ml)	median (IQR)	1718 (2428)	1924 (1445)	0.01 (64.4)	<0.001
Duration of symptoms (days)	median (IQR)	7 (6)	3 (5)	8 (6)	<0.001
Discharge from ED	*n* (%)	19 (19)	9 (11.7)	10 (43.5)	0.001
Admission general ward	*n* (%)	81 (81)	68 (88.3)	13 (56.5)	0.001
Admission ICU	*n* (%)	20 (20)	20 (26)	0 (0)	<0.001
Mortality	*n* (%)	7 (7)	7 (9)	0 (0)	<0.001

Abbreviations: CRP, C‐reactive protein; ED, emergency department; ICU, intensive care unit; LDH, lactate dehydrogenase; MxA, myxovirus resistance protein‐1.

**Figure 2:**
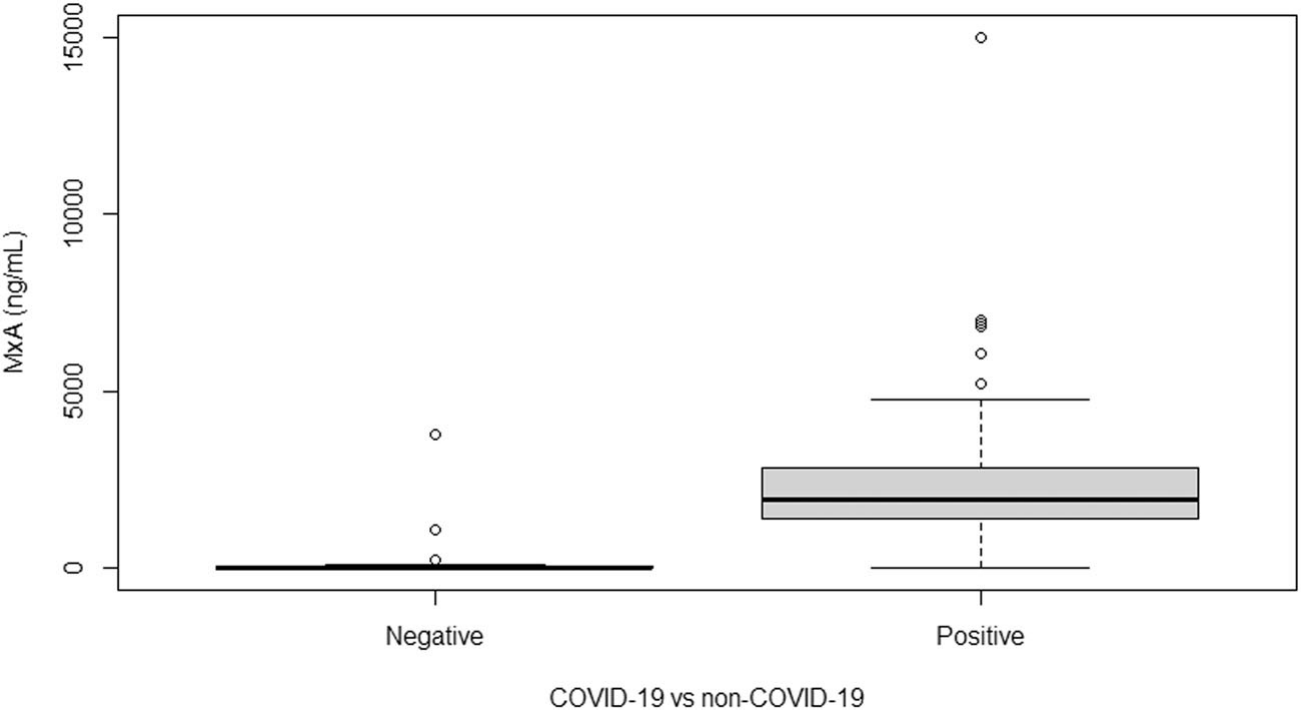
Boxplot of MxA levels in COVID‐19‐negative and ‐positive patients. MxA, myxovirus resistance protein‐1

**Figure 3:**
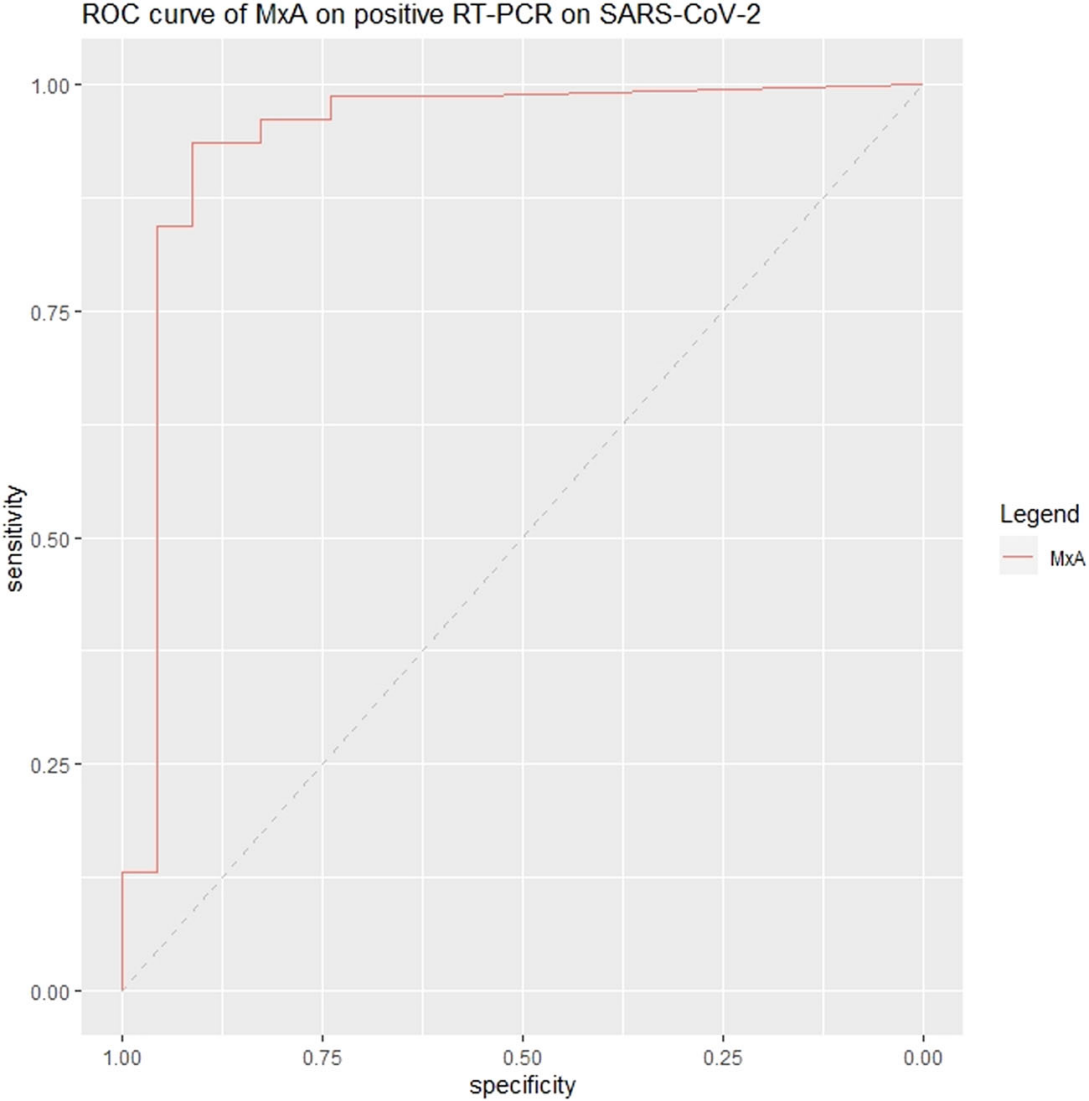
ROC curve of MxA. MxA, myxovirus resistance protein‐1; ROC, receiver operating chart; RT‐PCR, reverse‐transcriptase‐polymerase chain reaction; SARS‐COV‐2, severe acute respiratory syndrome coronavirus 2

Median MxA level was significantly higher (*p* < .001) in COVID‐19 patients compared to non‐COVID‐19 patients, respectively 1933 and 0.1 ng/ml (Figure 2). The AUC of MxA on a confirmed COVID‐19 infection was 0.941 (95% CI: 0.867–1.000) (Figure 3). Using Youden's Index, the optimal cut‐off point of MxA was 252 ng/ml. At this cut‐off point, the sensitivity of MxA on a confirmed COVID‐19 infection was 94% (95% CI: 85%–98%) and the specificity was 91% (95% CI: 72%–99%) (Table 2).

**Table 2:** Sensitivity, specificity, negative predictive value, and positive predictive value of MxA at different cut‐off values (percentages) (see File S1)

MxA cut‐off value	Sensitivity	Specificity	Negative predictive value	Positive predictive value
20 ng/ml	99 (93–100)	61 (39–80)	93 (68–100)	89 (81–95)
252 ng/ml	94 (85–98)	91 (72–99)	81 (61–93)	97 (91–100)
434 ng/ml	92 (84–97)	91 (72–99)	78 (58–91)	97 (90–100)

Abbreviation: MxA, myxovirus resistance protein‐1.

Using a predefined cut‐off point of 20 ng/ml,^12^ the sensitivity of MxA on a confirmed COVID‐19 infection was 99% (95% CI: 93%–100%) with a specificity of 61% (95% CI: 39%–80%). At a cut‐off point of 434 ng/ml, the sensitivity of MxA was 92% (95% CI: 84%–97%), and the specificity was 91% (95% CI: 72%–99%).

The characteristics of patients using immunosuppressive medication and immunodeficiency are shown in File S2. There was no significant difference (*p* = .904) in median MxA level in patients using immunosuppressive medication (*n* = 46) with a median of 1702 versus 1760 ng/ml in patients not using immunosuppressive medication. The median MxA level in patients with immunodeficiency (*n* = 11) was 932 versus 1756 ng/ml in patients without an immunodeficiency, which was not significantly different (*p* = .292).

## 4 DISCUSSION

In this study, we investigated the diagnostic value of MxA and found an AUC of 0.941 (95% CI: 0.867–1.000) on identifying COVID‐19 infections in patients presenting with suspected COVID‐19 infection in the ED. Based on these results, patients with COVID‐19 can be accurately identified when they have an elevated MxA level in the ED.

MxA has previously been investigated as biomarker of other viral infections in the ED, such as influenza.^7^, ^11^ Furthermore, MxA has been incorporated as point‐of‐care‐test FebriDx, which was developed as a triage tool to quickly rule out or rule in viral infections.^12^ In COVID‐19 patients, MxA has been investigated by several studies.^13^, ^14^ A study by Lagi et al.^15^ investigated FebriDx in hospitalized patients and found a sensitivity of 97.8% and specificity of 95.3% on a confirmed COVID‐19 infection. The cut‐off point of MxA in the FebriDx test is 20 ng/ml, which is lower than the optimal cut‐off point we found in our study. Although the MxA assay we used differs from the FebriDx test, we also tested MxA with a cut‐off of 20 ng/ml and found a similar sensitivity, but lower specificity. This difference can be explained by the different settings of the studies. The study of Lagi et al.^15^ included patients which were already diagnosed with COVID‐19 or an alternative diagnosis in the hospital's infectious wards. In contrast, in our study, patients were included before the final diagnosis was made. Therefore, our results are applicable to the ED setting, where a final diagnosis often is yet to be determined. A study by Karim et al. was conducted in a setting similar to ours and reported a sensitivity of 85% and specificity of 100% using FebriDx in the ED in patients with a suspected COVID‐19 infection.^13^ The current method of measuring MxA levels in whole blood requires 2‐h incubation, reducing its potential for use in the ED. However, faster methods of measuring MxA are available.^16^ Based on our findings, MxA may improve the diagnostic workup in suspected COVID‐19 patients in the ED. Implementing MxA point‐of‐care‐testing as regular laboratory measurement in the ED can speed up decisions on required patient isolation measures before SARS‐CoV‐2 RT‐PCR results are available.^17^ More specifically, in case of low MxA levels patients, in our study below 252 ng/ml, patients may not need to be isolated, whereas patients with an elevated MxA require quarantine measures until the RT‐PCR results are available. Such an approach could reduce the burden on the, often limited, examination rooms with isolation or quarantine functions in the ED. Furthermore, when patients have a probable viral infection with a high MxA, the treating physician may consider withholding antibiotic treatment until a definite diagnosis is made. MxA could also be investigated as triage tool to guide treatment and isolation decisions in patients with a suspected respiratory tract infection in the ED.

MxA is upregulated by type‐I interferons in response to viral infection. Patients using immunosuppressive medication or patients with immunodeficiency may have a reduced type I interferon activity and consequently diminished MxA production.^18^ Studies on the clinical use of MxA as a potential biomarker often exclude patients using immunosuppressive medication. Our study included 100 patients of which a total of 11 had a form of immunodeficiency while another 46 used immunosuppressive medication. We did not find a significant difference in MxA levels between patients with or without immunosuppressive medication and patients with or without any form of immunodeficiency. This suggests that MxA synthesis in the case of SARS‐CoV‐2 infection is not suppressed under these conditions. However, the study was not powered to detect such a difference and the group of patients with immunodeficiency was small (*n* = 11). These findings may be at risk of a Type 2 error and further studies powered to find a difference in MxA levels in these groups should be performed to validate these findings.

## 5 LIMITATIONS

Our study has several limitations. Our study was conducted during the COVID‐19 pandemic when the SARS‐CoV‐2 prevalence was high. MxA is a mediator in antiviral immune responses in general, and does not specifically reflect the host response to COVID‐19 infection, but also the host response to other respiratory tract infections.^19^ The findings of our study may not simply be applicable to patient populations when the prevalence of viral respiratory tract infections is different.^20^ Furthermore, the group of non‐COVID‐19 patients consisted only of patients with a bacterial infection or noninfectious diagnosis. Therefore, our results can only be interpreted as MxA being able to distinguish COVID‐19 patients from patients with bacterial infections or no infection. We hypothesize that MxA will show similar results in seasons where other viruses, such as influenza, are more prevalent, but validation in these seasons is required.

The study population was relatively small with 100 patients, included in an academic hospital. These results require international multicenter validation. Furthermore, informed consent needed to be obtained before enrolling patients in this study. Often, critically ill patients or patients with severe respiratory symptoms were not able to give informed consent. Therefore, there may have been a selection bias towards noninclusion of this group of patients. We recommend that MxA is thoroughly validated in critically ill patients. Lastly, our study enrolled patients with a clinical suspected COVID‐19 infection. Therefore, these results are not validated in asymptomatic patients or patients that visit the ED for other reasons than a suspected COVID‐19 infection.

## 6 CONCLUSION

Whole blood MxA levels accurately distinguish COVID‐19 infections from bacterial infections and noninfectious diagnoses in the ED in patients with a suspected COVID‐19 infection. The results of this study indicate that MxA measurements may be of added value in the diagnostic workup and patient flow in the ED. Validation of these findings is recommended before implementation in routine clinical practice is issued.

## CONFLICTS OF INTEREST

The authors declare no conflicts of interest.

## AUTHOR CONTRIBUTIONS

Kirby Tong‐Minh, Samantha van Hooijdonk, Virgil A. S. H. Dalm, and Willem A. Dik were involved in the conception or design of the manuscript. Kirby Tong‐Minh, Samantha van Hooijdonk, and Yuri van der Does performed the analysis and interpretation of the data. Kirby Tong‐Minh and Samantha van Hooijdonk drafted the manuscript. Kirby Tong‐Minh, Samantha van Hooijdonk, Marjan A. Versnel, Cornelia G. van Helden‐Meeuwsen, Petrus Martin van Hagen, Eric C. M. van Gorp, Henrik Endeman, Yuri van der Does, Virgil A. S. H. Dalm, and Willem A. Dik were involved in the critical revision of the manuscript and final approval of the manuscript.

## ETHICS STATEMENT

The study was approved by the local institutional review board and registered under number: NL73846.078.20. This study was conducted in accordance with the Declaration of Helsinki (64th WMA General Assembly, Fortaleza, Brazil, October 2013).

## Supporting information

Supplementary information.

Supplementary information.

## Data Availability

The datasets used and/or analyzed during the current study are available from the corresponding author on reasonable request.
