# Supplementary material for: Blood myxovirus resistance protein‐1 measurement in the diagnostic work‐up of suspected COVID‐19 infection in the emergency department
Source: Immun Inflamm Dis. 2022 Mar 29;10(4):e609. doi: 10.1002/iid3.609 (PMC8962640; doi:10.1002/iid3.609)
Supplement: Supplementary file 2 — Supplementary information. [file IID3-10-0-s002.docx]

**Additional File 1**

Additional Table 1: Patient characteristics of patients with and without immunodeficiency.

| **Patient characteristics** |  | **All patients** |  | **Immunodeficieny** |  | **No immunodeficiency** |  | **p-value** |
| --- | --- | --- | --- | --- | --- | --- | --- | --- |
|  |  | n=100 |  | n=89 |  | n=11 |  |  |
| **Demograpic data** |  |  |  |  |  |  |  |  |
| Gender: male | n (%) | 62 (62) |  | 58 (65) |  | 4 (36) |  | 0.127 |
| Age | Median (IQR) | 59.0 (24.0) |  | 60.0 (24.0) |  | 47.0 (23.0) |  | 0.0698 |
|  |  |  |  |  |  |  |  |  |
| **Vital parameters** |  |  |  |  |  |  |  |  |
| Heartrate (/min) | mean (SD) | 86.1 (15.8) |  | 83.9 (13.9) |  | 104 (18.4) |  | 0.00402 |
| Respiratory rate (/min) | median (IQR) | 18.0 (7.25) |  | 18.0 (7.00) |  | 18.0 (7.00) |  | 0.605 |
| Oxygen saturation (%) | median (IQR) | 96.0 (3.00) |  | 96.0 (3.00) |  | 95.0 (2.00) |  | 0.341 |
| Diastolic blood pressure (mmHg) | mean (SD) | 77.9 (11.1) |  | 77.9 (11.3) |  | 78.0 (9.80) |  | 0.967 |
| Systolic blood pressure (mmHg) | mean (SD) | 132 (17.2) |  | 132 (17.7) |  | 135 (13.7) |  | 0.564 |
| Temperature (Celsius) | mean (SD) | 37.6 (1.02) |  | 37.5 (0.968) |  | 38.5 (1.13) |  | 0.0226 |
|  |  |  |  |  |  |  |  |  |
|  |  |  |  |  |  |  |  |  |
| **Laboratory testing** |  |  |  |  |  |  |  |  |
| Procalcitonin (ng/mL) | median (IQR) | 0.120 (0.188) |  | 0.120 (0.178) |  | 0.140 (0.280) |  | 0.471 |
| CRP (mg/L) | median (IQR) | 62.5 (98.0) |  | 59.0 (94.0) |  | 114 (113) |  | 0.678 |
| Leucocyte count | median (IQR) | 6.40 (4.23) |  | 6.40 (3.90) |  | 6.30 (4.45) |  | 0.37 |
| Ferritine (ug/mL) | median (IQR) | 677 (855) |  | 729 (914) |  | 354 (462) |  | <0.001 |
| LDH (U/L) | median (IQR) | 316 (139) |  | 320 (141) |  | 262 (100) |  | 0.16 |
| Lactate (mmol/L) | median (IQR) | 1.30 (0.800) |  | 1.40 (0.950) |  | 0.900 (0.300) |  | 0.143 |
|  |  |  |  |  |  |  |  |  |
| MxA (ng/mL) | median (IQR) | 1718 (2428) |  | 1924 (1445) |  | 0.01 (64.4) |  | <0.001 |

Additional Table 2: Patient characteristics of patients using immunosuppressive medication and not using immunosuppressive medication.

| **Patient characteristics** |  | **All patients** |  | **Immunosuppresive medication** |  | **No immunosuppresive medication** |  | **p-value** |
| --- | --- | --- | --- | --- | --- | --- | --- | --- |
|  |  | n=100 |  | n=46 |  | n=54 |  |  |
| **Demograpic data** |  |  |  |  |  |  |  |  |
| Gender: male | n (%) | 62 (62) |  | 26 (57) |  | 36 (67) |  | 0.404 |
| Age | Median (IQR) | 59.0 (24.0) |  | 61.5 (18.8) |  | 55.5 (26.5) |  | 0.107 |
|  |  |  |  |  |  |  |  |  |
| **Vital parameters** |  |  |  |  |  |  |  |  |
| Heartrate (/min) | mean (SD) | 86.1 (15.8) |  | 85.8 (13.8) |  | 86.5 (18.0) |  | 0.839 |
| Respiratory rate (/min) | median (IQR) | 18.0 (7.25) |  | 18.0 (7.00) |  | 18.0 (5.50) |  | 0.93 |
| Oxygen saturation (%) | median (IQR) | 96.0 (3.00) |  | 96.0 (2.75) |  | 95.0 (3.00) |  | 0.28 |
| Diastolic blood pressure (mmHg) | mean (SD) | 77.9 (11.1) |  | 77.2 (10.6) |  | 78.7 (11.8) |  | 0.528 |
| Systolic blood pressure (mmHg) | mean (SD) | 132 (17.2) |  | 131 (17.9) |  | 134 (16.4) |  | 0.5640.287 |
| Temperature (Celsius) | mean (SD) | 37.6 (1.02) |  | 37.7 (1.09) |  | 37.6 (0.948) |  | 0.607 |
|  |  |  |  |  |  |  |  |  |
|  |  |  |  |  |  |  |  |  |
| **Laboratory testing** |  |  |  |  |  |  |  |  |
| Procalcitonin (ng/mL) | median (IQR) | 0.120 (0.188) |  | 0.130 (0.330) |  | 0.120 (0.155) |  | 0.618 |
| CRP (mg/L) | median (IQR) | 62.5 (98.0) |  | 66.0 (115) |  | 60.0 (96.8) |  | 0.209 |
| Leucocyte count | median (IQR) | 6.40 (4.23) |  | 6.50 (3.83) |  | 6.35 (4.60) |  | 0.906 |
| Ferritine (ug/mL) | median (IQR) | 6.40 (4.23) |  | 6.50 (3.83) |  | 6.35 (4.60) |  | 0.618 |
| LDH (U/L) | median (IQR) | 316 (139) |  | 312 (160) |  | 319 (128) |  | 0.459 |
| Lactate (mmol/L) | median (IQR) | 1.30 (0.800) |  | 1.40 (1.15) |  | 1.20 (0.550) |  | 0.132 |
|  |  |  |  |  |  |  |  |  |
